# Malignancy without immortality? Cellular immortalization as a possible late event in melanoma progression

**DOI:** 10.1111/j.1755-148X.2011.00850.x

**Published:** 2011-03-21

**Authors:** Julia K Soo, Alastair D MacKenzie Ross, David M Kallenberg, Carla Milagre, W Heung Chong, Jade Chow, Lucy Hill, Stacey Hoare, Rebecca S Collinson, Mehnaz Hossain, W Nicol Keith, Richard Marais, Dorothy C Bennett

**Affiliations:** 1 Division of Biomedical Sciences, St. George's, University of LondonUK; 2 Institute of Cancer ResearchLondon, UK; 3 Division of Cellular and Molecular Medicine, St. George's, University of LondonUK; 4 South West Thames Regional Cytogenetics Service, St. George's NHS TrustLondon, UK; 5 Institute of Cancer Sciences, Cancer Research UK Beatson Laboratories, University of GlasgowGlasgow, UK

**Keywords:** cell senescence, crisis, immortalization, primary melanoma, radial growth-phase, nevus

## Abstract

Cell senescence is a permanent growth arrest following extended proliferation. Cultured cancer cells including metastatic melanoma cells often appear immortal (proliferate indefinitely), while uncultured benign nevi (moles) show senescence markers. Here, with new explantation methods, we investigated which classes of primary pigmented lesions are typically immortal. Nevi yielded a few proliferating cells, consistent with most nevus cells being senescent. No nevus culture (0/28) appeared immortal. Some thin and thick melanoma cultures proved immortal under these conditions, but surprisingly few (4/37). All arrested cultures displayed three senescence markers in some cells: β-galactosidase, nuclear p16, and heterochromatic foci/aggregates. However, melanoma cultures also showed features of telomeric crisis (arrest because of ultrashort telomeres). Moreover, crisis markers including anaphase bridges were frequent in uncultured vertical growth-phase (VGP) melanomas. Conversely, all immortal melanoma cultures expressed telomerase reverse transcriptase and telomerase, showing aneuploidy. The findings suggest that primary melanomas are typically precrisis, with immortalization/telomere maintenance as a late event.

## Introduction

Cell senescence, a form of permanent growth arrest, has emerged as a central mechanism of tumor suppression and a potential source of therapeutic targets for diverse malignancies. Normal mammalian somatic cells enter senescence following either extensive proliferation (replicative senescence), activation of an oncogene (oncogene-induced senescence), or cellular stresses including oxidative or DNA damage (d’Adda di Fagagna, 2008; Collado et al., 2005; Mooi and Peeper, 2006; Stewart and Weinberg, 2006). Cells of various kinds of benign, static neoplasms in vivo show markers of senescence, for example, in melanocytic nevi, which generally carry activating v-*raf* murine sarcoma viral oncogene homolog B1 (*BRAF)* or neuroblastoma *RAS* viral oncogene homolog (*NRAS)* mutations (Gray-Schopfer et al., 2006; Michaloglou et al., 2005). It is proposed that clonal proliferation initiated by an oncogene is normally limited by oncogene-induced senescence, giving a benign lesion rather than a growing cancer (Bennett and Medrano, 2002; Feldser and Greider, 2007; Gray-Schopfer et al., 2006; Michaloglou et al., 2005; Mooi and Peeper, 2002). Moreover, in human cells, escape from senescence usually leads to a further kind of arrest called telomeric crisis, another barrier to immortality (Artandi and DePinho, 2010; Bond et al., 1999). Melanoma, now the sixth commonest cancer in the UK following progressive increases in incidence and mortality (CR-UK Statistical Information Team, 2010), is an amenable research model for senescence, crisis, and immortalization in cancer progression, as early melanomas and their potential precursor lesions, nevi, are accessible and are treated by excision. The main aim of this study was to find out at which stage in melanoma progression melanocytes typically become immortal.

**Significance**

Cell senescence is a major tumor suppressor mechanism and a target of familial melanoma genes, while conversely, immortality is widely considered a hallmark of cancer. However, it is unclear whether early cancers are immortal, because most cultured lines arise from advanced cancers. We tested this using improved culture conditions and found unexpectedly that many primary melanomas, despite growing well, eventually arrested. This difference from the previously reported behavior of many metastatic melanomas suggests prognostic value for markers of immortality. We also observed that cells from radial growth-phase (RGP) melanomas consistently require keratinocyte products for survival, potentially explaining their thin form.

Several signaling pathways can mediate cell senescence (reviews: Cichowski and Hahn, 2008; d’Adda di Fagagna, 2008). Telomere shortening is strongly implicated in human replicative senescence: DNA-damage signaling from short telomeres activates p53, transcriptionally upregulating the cyclin-dependent kinase inhibitor (CDKI) p21 (d’Adda di Fagagna, 2008; Collado et al., 2007; Herbig and Sedivy, 2006). Replicative and oncogene-induced senescence also often involve another major tumor suppressor and CDKI, p16 [inhibitor A of kinase 4 (INK4A)] (Bond et al., 1999; Herbig and Sedivy, 2006; Jacobs and de Lange, 2005; Serrano et al., 1997). p16 upregulation can involve BMI1 (Itahana et al., 2003) and/or JMJD3 (Barradas et al., 2009). p16 affects permanent cell arrest by inhibiting cyclin-dependent kinase 4 (CDK4) and CDK6 and thereby activating the retinoblastoma (RB) family of growth inhibitors.

p16 is encoded at the locus most commonly mutated in familial melanoma, *CDKN2A*, underlining its importance in melanoma (Bennett, 2008; Gray-Schopfer and Bennett, 2006; Ha et al., 2008; Mooi and Peeper, 2006). *CDKN2A* also encodes another protein, alternative reading frame (ARF), another effector of senescence (Ha et al., 2008), although not clearly so in human cells (Wei et al., 2001). Melanoma-associated mutations of p16 were confirmed to disable cell senescence in melanoma cells (Haferkamp et al., 2008).

It is generally proposed that cellular immortalization (acquired ability to divide indefinitely) is required for cancer development (Bennett, 2003; Collado et al., 2007; Mooi and Peeper, 2006; Stewart and Weinberg, 2006). However, this has not been proven for all types of cancer, especially early cancers. In the case of melanoma, many immortal cell lines have been derived from metastatic melanomas, but fewer from primary melanomas (Herlyn et al., 1985a; Hsu et al., 2000; Pope et al., 1979; Semple et al., 1982). Primary cutaneous melanocytic lesions can be classified into four stages: benign nevi or moles, dysplastic nevi, radial growth-phase (RGP) melanomas, which grow in the epidermis with micro- or no invasion, and vertical growth-phase (VGP) melanomas, which invade the deeper dermis and appear competent for metastasis (Clark et al., 1989; Herlyn et al., 1985a).

The molecular requirements for immortalization specifically of human melanocytes have been established in recent years. As expected from its role in melanoma susceptibility, p16 appears important and defects in the p16/RB pathway in melanocytes lead to lifespan extension; however, the cells can then still become arrested, by a p53-dependent route (Sviderskaya et al., 2003). Human melanomas, while often retaining wild-type p53, may show downstream suppression of p53 signaling by various routes including TBX2 or DEK overexpression or LKB1/STK11 deficiency (Khodadoust et al., 2009); review: (Bennett, 2008). However, disruption of both the RB and p53 pathways is still not sufficient for immortalization. Human cells (including melanocytes) that evade the normal senescence pathways yet lack telomerase can resume division and telomere shortening but reach a further growth barrier called telomeric crisis. This can be defined as the state resulting in a cell population from loss of telomere function following extreme shortening (Artandi and DePinho, 2010; Chin et al., 2004). It is a stage of continuing cell division balanced by extensive cell death, as the unprotected chromosome ends can be ligated to each other by the DNA repair machinery. Ligated chromosomes lead to anaphase bridging [a dicentric chromosome(s) attached to both poles] and thence mitotic catastrophe, chromosomal breakages, and rearrangements (Artandi and DePinho, 2010; Bond et al., 1999; DePinho and Polyak, 2004; Gisselsson and Höglund, 2005). Telomeric crisis has been described as a stage in the progression of human breast, pancreatic, colorectal, and other cancers (Chin et al., 2004; Hezel et al., 2006; Höglund et al., 2003; Rudolph et al., 2001). Escape from telomeric crisis, in culture or in cancer development, requires telomere maintenance, which permits immortality and which is usually achieved, either as a rare natural event (in cancers) or artificially, by upregulated expression of telomerase reverse transcriptase (TERT), the catalytic subunit of telomerase (Bennett, 2008; Bond et al., 1999; DePinho and Polyak, 2004; Newbold, 2002; Wynford-Thomas, 1999). Telomere maintenance combined with a defect in the p16/RB pathway appears necessary and sufficient for full immortalization of human melanocytes (Gray-Schopfer et al., 2006; Sviderskaya et al., 2003).

We became interested to determine whether immortality arises at a particular stage in melanoma progression. There is some information on the acquisition of the required changes, RB lesions, and telomerase activation. p16 expression appears typically strong in benign nevi, reduced in dysplastic nevi and RGP melanomas, and lost (as nuclear p16) in VGP melanomas (Gray-Schopfer et al., 2006; Keller-Melchior et al., 1998; Talve et al., 1997). Several groups report high telomerase activity in cells from metastatic melanomas, but reports differ regarding telomerase activity in primary lesions (Carvalho et al., 2006; Glaessl et al., 1999; Rudolph et al., 2000). We previously proposed a model in which benign nevi show oncogene-induced senescence and melanoma is initiated by immortalization, through p16/RB deficiency and telomerase expression (Bennett, 2003; Gray-Schopfer and Bennett, 2006). Marker studies are consistent with this (Gray-Schopfer et al., 2006; Michaloglou et al., 2005) but have not definitively shown which lesions are immortal; thus, the idea that nevi are senescent has been questioned (Cotter et al., 2007). Previous culture studies have not given definitive answers regarding which pigmented lesions are immortal. Proliferating cells in nevus cultures (Halaban et al., 1986; Mancianti et al., 1988) might have been normal melanocytes, as these were not excluded, and in descriptions of cultured RGP and VGP melanoma cells (e.g. Herlyn et al., 1985b; Hsu et al., 2000), only established lines were well characterized, leaving it unclear whether other cultures arrested. Explantation with extended cell culture is the best available routine assay to test for immortality, because even once established as immortal cultured lines, RGP and early VGP cells are usually poorly or non-tumorigenic in immunodeficient mice (Hsu et al., 2000; Kobayashi et al., 1994).

Thus, to test explicitly at what clinical stage(s) in melanoma development immortality appears, we have made new primary cultures to study culture lifespan (though this is slow and laborious; this report summarizes over 4 years’ work.) Re-examining growth requirements, we developed conditions in which cells of all four lesion types showed good initial viability and growth. In summary, despite this initial growth, no benign or dysplastic nevus culture was immortal, and surprisingly, only a minority of primary melanoma cultures were immortal under these conditions. Combined data from their phenotypes and in vivo markers suggest that arrest of melanoma cultures reflected telomeric crisis rather than senescence.

## Results

### Favorable culture conditions for typical melanocytic lesional cells

**Figure 1:**
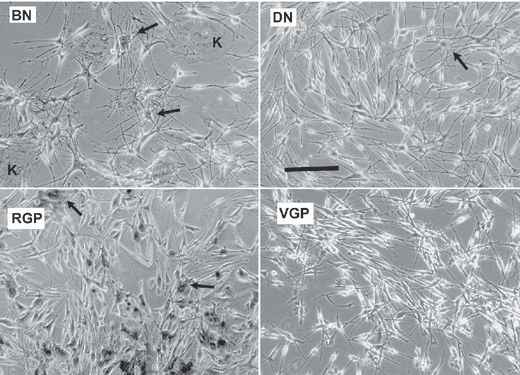
Initial morphology of cells from different stages of progression. Phase-contrast images of cells of each lesion type at low passage levels. BN: a benign nevus at passage 1 (primary explant culture being passage 0), showing highly dendritic cells (arrows). K: keratinocyte feeder cells. DN: dysplastic nevus, passage 1. Polydendritic cells (arrows) as well as bipolar and oligodendritic cells. RGP: radial growth-phase melanoma (SGM3), passage 1. Large, well-pigmented cells were present in this example (arrows). VGP: vertical growth-phase melanoma (SGM5), passage 3. Typically cells were smaller than for other lesion types and bipolar or tripolar. Quite similar to normal growing melanocytes but dendrites wider. Scale bar for all panels: 150 μm.

Melanomas and dysplastic nevi are frequently deficient in the p16/RB pathway (Gray-Schopfer et al., 2006). Therefore, the initial culture conditions tested were adapted from those developed for p16-deficient melanocytes, which have a high apoptotic tendency and require either keratinocytes or keratinocyte-derived factors for survival (Sviderskaya et al., 2003). Features included mitomycin-inactivated mouse keratinocyte feeder cells and a neutral pH medium containing fetal calf serum (FCS), 12-*O*-tetradecanoyl phorbol 13-acetate (TPA), cholera toxin (CT), and stem cell factor (SCF), which is secreted by human keratinocytes. Despite the very small size of samples, estimated to yield typically around 10^3^–10^5^ viable lesional cells in all, these conditions yielded healthy appearing melanocytic cells from nearly all samples of all lesion types (Figure 1). These were presumably virtually all lesional cells, as the epidermis with normal melanocytes was excluded (or cultured separately). Fibroblast contamination was rare; cultures with fibroblasts were discarded and not analyzed here, as they overgrew the lesional cells. Some keratinocytes attached initially from epidermal explants but did not proliferate in this medium.

The appearance of the cultures was very variable, but differences between lesion types emerged. Benign and dysplastic nevus cells often resembled senescent cultured melanocytes: large, non-dividing, flat, or multidendritic cells (Figure 1). Other nevus cultures initially yielded some growing melanocytes (bi- or tripolar), which acquired a senescent appearance only after one or more passages (Figure S1). Initial growth was, however, often in the form of colonies, suggesting that only a small fraction of explanted cells were capable of proliferation. Senescent cultured melanocytes often attach poorly after subculture, so this may likewise be true of senescent cells ex vivo.

**Figure 2:**
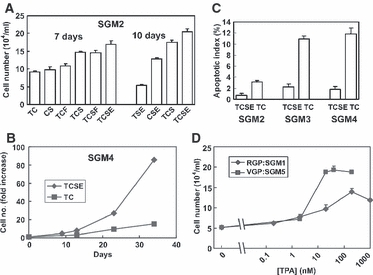
Factor dependence retained by radial growth-phase (RGP) melanoma cells. Key: C: CT, 200 pM. E: EDN1, 10 nM. F: XB2 feeder cells. S: SCF, 10 ng/ml. T: TPA, 200 nM. (A) Response to combinations of mitogens. SGM2 RGP cells at passage 15 were plated at 3 × 10^4^ cells/ml with the indicated combinations of factors and grown for 7 or 10 days as indicated (two separate experiments), with medium changes every 3–4 days. Cells were harvested and counted; means and SEM of triplicate dishes are shown. (B) Longer time course of response to SCF and EDN1. SGM4 cells were grown with and without these factors for several passages. Triplicate cultures were counted after each passage and the fold increase was calculated. These were multiplied to obtain total fold increase. Substantially better growth with SCF and EDN1. Similar results were obtained with RGP line SGM3, except this line grew more slowly under both conditions. (C) High apoptotic rates of three RGP lines (SGM2-4), suppressed by stem cell factor (SCF) and EDN1. Cells were grown for about 5 weeks with and without SCF and EDN1, and then the TUNEL method was used to assess % apoptotic cells (Methods). Means and SEM of triplicate cultures are shown. (D) Dose-response of cell numbers to TPA in typical RGP and vertical growth-phase (VGP) cultures. RGP cells (SGM1) or VGP cells (SGM5) were plated at 3 × 10^4^/ml and grown for 7 days with SCF, cholera toxin, and the stated amounts of 12-*O*-tetradecanoyl phorbol 13-acetate (TPA). Means and SEM of triplicate counts are shown. Note that SGM5 cells reached higher densities than other strains; so for this line, the RPMI 1640 medium was mixed 50:50 with DMEM to avoid acidification. Counts for cultures with 20 nM TPA gave no significant difference between this mixture and 100% RPMI 1640 at day 7 (not shown).

Melanoma cultures initially tended to have fewer senescent-looking cells (Figure 1). Preliminary experiments suggested that CT and SCF promoted the growth of nevus and RGP melanoma cultures and that 200 nM TPA was an optimal level, just as for normal melanocytes (Bennett et al., 1985, 1987). Cultures were therefore provisionally grown and passaged in the initial medium, giving stocks for quantitative experiments, which gave similar results. Figure 2A shows two typical experiments with an RGP culture, indicating higher net growth with SCF as well as TPA and CT, slightly further improved by endothelin 1 (EDN1), another keratinocyte product. EDN1 was not, however, routinely added at explantation because it is also a mitogen for fibroblasts. Omission of either TPA or CT also reduced net growth. Keratinocyte feeder cells did not improve growth compared to the four factors (at the tested cell densities). Longer-term growth curves for two RGP cultures showed markedly more growth with the four supplements compared to TPA + CT only (Figure 2B and data not shown). RGP melanoma cells consistently showed substantial rates of apoptosis in the absence of SCF and EDN1 (Figure 2C), as seen previously with p16-deficient but not normal melanocytes (Sviderskaya et al., 2003). (This is consistent with the known frequent defects of the p16/RB pathway in melanoma cells.) RGP cells gave biphasic dose–response curves to TPA, with 200 nM generally optimal for growth (Figure 2D), as with normal human and mouse melanocytes (Bennett et al., 1985, 1987). While some RGP cells grew equally well with 20 nM TPA (data not shown), 200 nM TPA was adopted as routine. We thus retained the initial tested conditions for routine nevus and RGP cultures. VGP cell growth, however, appeared best with 20–40 nM TPA (Figure 2D), so 40 (later 20) nM TPA was adopted for VGP cultures.

### Culture lifespan and cell senescence

**Figure 3:**
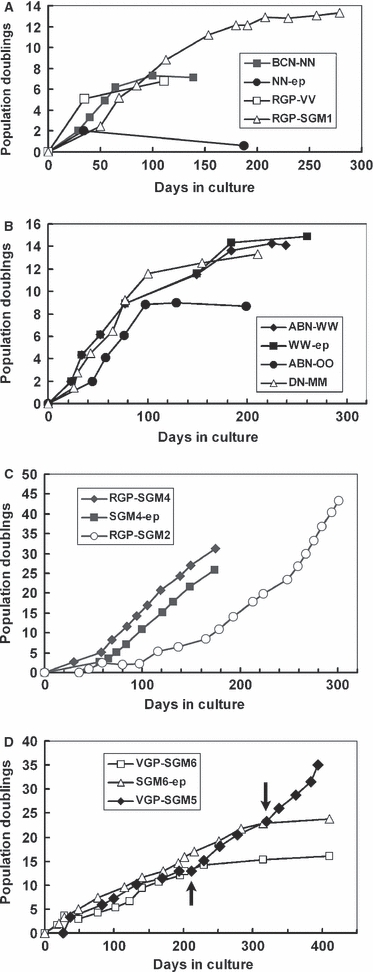
Types of growth patterns for nevus and primary melanoma cultures. Cultures that grew sufficiently to be subcultured were counted at each subculture (Methods). The fold increase for each passage was calculated and converted to cumulative doublings from the first subculture; each point marks one passage. Dermal-only fractions of lesions are shown, except (ep): epidermal fraction of same lesion. Key: BCN, benign compound nevus; ABN, atypical benign nevus (no dysplasia); and DN, dysplastic nevus. (A, B) Initial growth followed by slowing and arrest – the most common pattern. (C) Initially slow growth followed by acceleration, seen only in immortal melanoma cultures (here radial growth-phase lines SGM2 and SGM4). (D) Deceleration followed by acceleration steps (arrows), in VGP culture SGM5. The first acceleration followed reduction of the 12-*O*-tetradecanoyl phorbol 13-acetate (TPA) concentration to 20 nM on obtaining of dose–response data; the second was spontaneous. Arrest of both portions of VGP culture SGM6 (despite reduced TPA) also shown.

Growth curves were derived from cell counts over the lifespan of each culture (or for a year or more) to identify senescence or immortality. Some cultures failed to grow sufficiently for any subculture, while others were subcultured. Various types of growth kinetics were observed: most commonly proliferation and then arrest (Figure 3A,B and D), alternatively slow growth and then acceleration (Figure 3C), or deceleration and then acceleration [St. George's melanoma (line) 2 (SGM2) in Figure 3C]. Acceleration suggests ‘progression’ in culture – the acquisition of additional genetic or epigenetic changes facilitating growth. It may also represent selection, for example, the epidermal portion of line SGM4 accelerated a few weeks after the dermal portion, suggesting that immortal cells were already present in both portions at explantation, and took over both cultures.

Table 1(A) shows the aggregated culture-lifespan data for lesions of all types. In summary, it can be seen that no benign or dysplastic nevus cultures showed numerous doublings nor immortality. RGP and VGP melanoma cultures proliferated for a wide range of different periods up to about 6 months but then (surprisingly) also typically became arrested. Arrested cells tended to become large and flat or multidendritic. Only about one in nine cultures of primary melanoma cells continued to proliferate indefinitely (for at least a year) without senescence and was thus taken to be immortal.

**Table 1:** Growth arrest in relation to senescence markers in cultures explanted from pigmented lesions. (A) Growth outcomes of cultures by lesion type. (B) Expression of senescence markers by all arrested cultures but not all cells

		Outcome (arrest or immortal)		
		
Diagnosis	Total successful attempts (i.e., pure melanocytes)	Did not grow enough to subculture (in at least 6 weeks)	Grew, subcultured, then stopped (2 weeks to 6 months)	Continues to grow – appears immortal
(A)				
Benign nevus	22^a^	7	15	0
Dysplastic/atypical nevus	6	4	2	0
RGP melanoma	22	11	8	3
VGP melanoma (reduced TPA)	15	6	7	1

	Proportion of arrested cultures positive^b^			Percentage of cells positive (mean, SEM)		
		
Diagnosis	β-galactosidase	p16	H3M3K9	β-galactosidase	p16	H3M3K9
(B)						
Nevus	12/12	14/14	12/12	65.8 ± 2.08	59.9 ± 3.30	76.6 ± 3.41
RGP melanoma	7/7	6/6 (fainter)	5/5	50.0 ± 5.12	61.7 ± 6.10	72.5 ± 13.14
VGP melanoma	11/11	8/8 (fainter)	8/8	22.2 ± 2.47	62.7 ± 5.73	77.3 ± 8.39

H3M3K9, histone H3 trimethylated on lysine 9; RGP, radial growth-phase; VGP, vertical growth-phase.

a Numbers of cultures in each category are shown. The data represent accumulated experience over 4 years. On pooling categories, there was a significant difference (P < 0.05) by the chi-squared test between the proportion of melanomas giving immortal cultures (5/37, 13.5%) compared with the proportion of nevi (0/28).

b Cultures that had arrested were trypsinized, plated on to sterile glass coverslips, and processed for cytochemistry (β-galactosidase) or by immunostaining for markers of cell senescence (Methods). For % cells positive, 100–200 cells per lesional culture were counted where possible, or otherwise as many as were visible. The percentage of cells positive may be an underestimate in some cases where staining may have been masked by melanin. Only a single dysplastic nevus culture had enough surviving cells at arrest for analysis; this was pooled with benign nevi. Intense fluorescence for H3M3K9 was counted as positive, whether or not multiple foci were distinguishable, as sometimes the foci were crowded and hard to distinguish, and intense fluorescence was not seen in immortal cultures. Among these arrested cultures, significantly fewer cells showed β-galactosidase staining in VGP than in nevus cultures (P = 4 × 10^−8^, very highly significant) and in VGP than in RGP cultures (P = 0.0012, significant) (2-tailed *t*-tests with Bonferroni multiple testing correction). Other differences were not significant, although p16 staining was visually judged to be typically fainter in melanoma than in nevus cultures.

**Figure 4:**
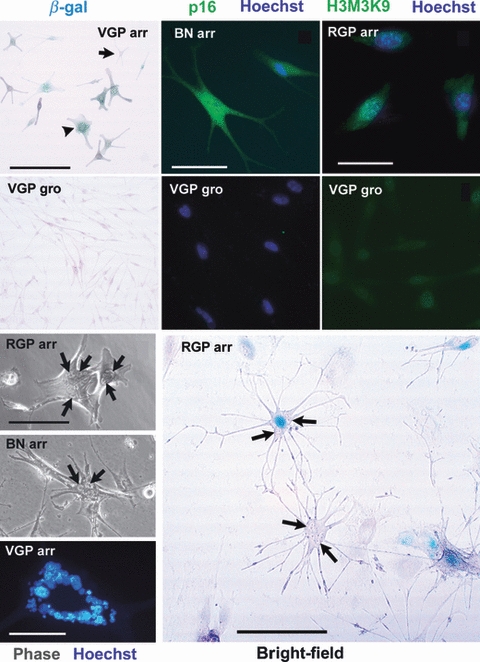
Expression of senescence markers and atypical morphology in arrested lesional cell cultures. Examples of marker-positive and negative cultures as quantitated in Table 1(B). BN, Benign nevus cells; (RGP, VGP) radial growth-phase and vertical growth-phase melanoma cultures. (arr, gro) Arrested and growing cultures. (β-gal, also bottom right image) Cultures stained for ‘senescence-associated’β-galactosidase (pH 7.0 as modified for melanocytes; Methods) (blue). Bars, 200 μm. Arrested VGP melanoma cultures typically showed cells ranging from small, faintly pigmented, and unstained (arrow, top left panel) to large, flat, well pigmented, and well stained (arrowhead). Growing cells typically negative. (p16/Hoescht) Cultures immunostained for p16 (FITC, green), counterstained with Hoechst 33258. Bar, 50 μm. The larger, flat, stellate cell has intense nuclear p16 masking the Hoechst 33258, while the other has mainly cytoplasmic p16. (Only nuclear p16 was considered positive.) Growing VGP cells were completely negative. (H3M3K9) Cultures immunostained for heterochromatin marker histone H3 trimethylated on lysine 9 (FITC, green). Bar, 50 μm. Green brightness digitally reduced in RGP panel to make senescence-associated heterochromatic foci (SAHF) sharper. SAHF are positive for both H3M3K9 and Hoechst, giving cyan color. Growing VGP culture shows no SAHF. Not counterstained, to show clearly the lack of foci and general paucity of heterochromatin. (Bottom 4 panels) Giant, multinucleate cells in arrested cultures. High-magnification images seen by phase contrast (gray; bar = 100 μm), Hoechst 33258 (blue fluorescence; bar = 20 μm) or bright-field optics with β-galactosidase stain (bar = 200 μm). Arrows indicate some of the micronuclei (pale), each with one or more nucleoli (dark). Cell debris and floating or poorly attached cells are also seen (phase-contrast images), indicative of cell death.

Where cultures arrested, the final cell yield was typically modest, about 10^2^–10^6^ cells, because starting numbers were low and the giant arrested cells did not reattach efficiently after subculture. Thus characterization generally had to involve small-scale methods like immunostaining. Arrested cultures were tested for three markers of cell senescence: p16, β-galactosidase, and senescence-associated heterochromatic foci (SAHF) (Narita et al., 2003) – multiple foci of nuclear heterochromatin containing trimethylated lysine 9 of histone H3 (H3M3K9). Examples are shown in Figure 4 and Figures S2 and S3. Note that melanoma nuclei are small and did not show distinct and separate SAHF as seen in fibroblasts, so cells with irregular masses of bright staining for H3M3K9 were also counted positive (Figure S3), as these were not generally seen in growing cultures. All arrested lesional cultures, whatever their origin, were positive for all three senescence markers (Table 1B), while tested immortal cultures were generally negative for all these markers (Figure 4). Not all cells within each arrested culture were detectably positive, however, although this is also true of normal senescent melanocyte cultures, in which 60–80% typically show senescence markers (data not shown). Likewise, senescent human fibroblasts showed between about 70–90% staining for β-galactosidase (Dimri et al., 1995). In nevus cells, p16 was often cytoplasmic as well as nuclear; this pattern seems common when expression levels are high, as also seen in nevi in vivo (Gray-Schopfer et al., 2006; Michaloglou et al., 2005). p16 fluorescence typically appeared less intense in arrested RGP and VGP than in nevus cultures and again was often cytoplasmic (Figure S2), while the percentage of cells positive for β-galactosidase declined significantly with progression stage, to only around 20% of cells in VGP cultures (Table 1B). The arrested RGP and VGP cultures also showed atypical morphology, with giant, multidendritic, and often multinucleate cells – also seen in nevus cultures but less commonly (Figure 4, lower panels) – and with noticeable cell death occurring. These features are not typical of normal senescence and suggested the possibility of telomeric crisis (where arrest represents continuing division balanced by death, and strong nuclear p16 expression would not be expected).

### Features of crisis in uncultured melanomas

**Figure 5:**
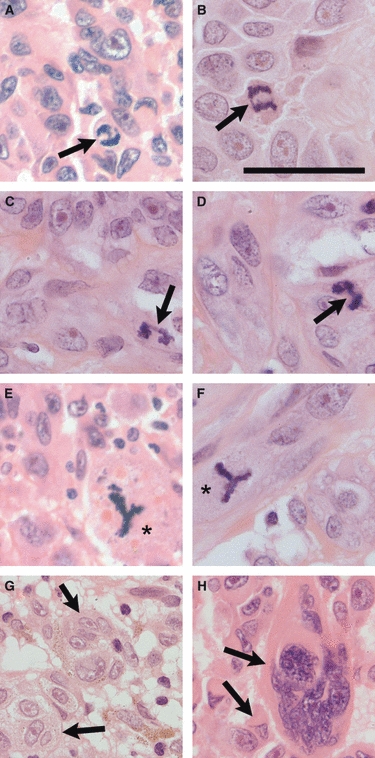
Markers of crisis in uncultured vertical growth-phase (VGP) melanomas. Sections of VGP melanomas from Cellular Pathology archival blocks, stained with hematoxylin and eosin. (A–D) Anaphase bridges (arrowed). Distinct bridge of chromatin between the two anaphase plates. (E–F) Tripolar mitoses, in giant cells (asterisks). (G–H) Multinucleate cells, arrowed. Bar for A–F, 25 μm; bar for G–H, 25 μm.

To test whether some primary melanomas may be at or approaching crisis, a series of uncultured melanoma sections was surveyed for anaphase bridges (dicentric chromosomes attached to both mitotic poles), a useful marker of crisis/telomere dysfunction (Artandi and DePinho, 2010; Chin et al., 2004; DePinho and Polyak, 2004; Gisselsson and Höglund, 2005; Gisselsson et al., 2001). Multinucleate cells and tripolar mitoses are also typical of crisis, although less specific, and these were also scored. All three features proved common in VGP melanomas (Figure 5; Table 2). Remarkably, over 21% of all anaphases in VGP lesions showed chromatin bridging. All three features were uncommon in RGP sections, but then few actual mitoses were observed here. This was consistent with textbook accounts that RGP melanomas lack mitoses in the dermal portion, while the epidermal portion is very thin (Mooi and Krausz, 2007), thus also providing few mitoses for analysis. At any rate, most VGP melanomas in vivo exhibit evidence of telomere dysfunction, in line with their lack of immortality in culture.

**Table 2:** Anaphase bridging and related abnormalities in primary VGP melanomas

Melanoma	Breslow thickness (mm) if > 1 mm	% anaphase bridges per anaphase	Tripolar mitoses observed	Multinucleate cells observed
1	9.5	0 (0/5)	Yes	Yes
2	9	31 (10/32)	Yes	Yes
3	6	14 (3/21)	Yes	Yes
4	3.4	20 (3/15)	Yes	Yes
5	3.2	0 (0/1)	No	Yes
6	2.3	16.7 (6/36)	Yes	Yes
7	2.2	26.5 (9/34)	Yes	Yes
8	2.1	30 (3/10)	Yes	Yes
9	1.96	0 (0/1)	No	Yes
10	1.6	19 (3/16)	Yes	Yes
11	1.4	? (0/0)	No	Yes
12		40 (2/5)	Yes	Yes
13		0 (0/5)	Yes	Yes
14		? (0/0)	Yes	Yes
15		? (0/0)	No	Yes
16		? (0/0)	No	Yes
17		? (0/0)	No	Yes

RGP, radial growth-phase; VGP, vertical growth-phase.

Anaphases and anaphase bridges were counted in hematoxylin- and eosin-stained sections of seventeen VGP primary melanomas from archived blocks at St. George's Healthcare Trust, ranked here by Breslow thickness. Frequency of bridging was high, totaling 21.5% of all anaphases seen. Anaphase bridges (Figure 5) were present in all VGP sections with over five mitoses. Tumors were also scored for the overall presence or absence of tripolar mitoses and multinucleate cells. The presence of tripolar mitoses correlated well with anaphase bridging, while multinucleate cells were observed in all melanomas. Six RGP melanomas were analyzed in the same way. However, percentage of bridging could not be determined because no anaphases were found in these sections.

### Long-lived and immortal cultures from early melanomas: chromosomal changes, oncogenes, and telomerase

Four new immortal lines and two long-lived cultures were obtained from primary melanomas. These were characterized by karyology, growth kinetics, and genotyping for the *BRAF* and *NRAS* mutations common in melanomas and nevi (Table 3). SGM1 was a long-lived culture from an RGP melanoma; these cells became arrested after about 6 months (Figure 3A), and no chromosomal abnormalities nor oncogene activation was detected. SGM2, SGM3, and SGM4 were RGP cultures that appeared immortal. SGM2 grew faster with increasing passage level, in at least two incremental steps (Figure 3C). SGM4 showed similar increments in growth rate after about 2 months, in both the epidermal and one dermal portion of the lesion (Figure 3C), suggesting that a small immortal subpopulation was present initially and grew out in both fractions. SGM5 was a VGP line that grew continuously, with acceleration at higher passage levels (Figure 3D). Lines SGM2 to SGM5 all had an activated allele of either *BRAF* or *NRAS* and chromosomal aberrations (Table 3), minor in SGM4 and SGM5. SGM6 was a VGP-derived culture that grew slowly, finally arresting after 23 doublings and 10 months. No mutation of *BRAF* or *NRAS* nor karyological change was detected in SGM6.

**Table 3:** Long-lived and immortal primary melanoma cultures: characteristics

Cell line	Source^a^	Oncogene	Karyotype	Shortest doubling time^b^ (days)
SGM1	RGP	None found	Diploid	9.2
SGM2	RGP	*BRAF* V600E	Subtetraploid: 85, XXX, −1, −3, −6, −9, −9, der(13,14)(q10;q10)	2.8^c^ (>passage 14)
SGM3	RGP	*BRAF* V600E	∼Tetraploid, translocations in 3p and 8q, unbalanced 3q and 4q^d^	4.9
SGM4	RGP	*BRAF* V600K	46, XX, add(7)(q?36), add(17)(q?25)	3.8
SGM5	VGP	*NRAS* Q61K	46, XY, der(9)t(9,13), +der(9)t(9,13), add(9)(p?24), −22	2.5
SGM6	VGP	None found	Diploid	6.3

*BRAF*, v-*raf* murine sarcoma viral oncogene homolog B1; RGP, radial growth-phase; SGM, St. George's melanoma (line); VGP, vertical growth-phase.

a Diagnosis based on sections adjacent to cultured portion.

b Calculated from steepest part of growth curve.

c or 5.3 days before passage 14.

d Details obscured by abundant pigment. Line SGM1 was long-lived but did arrest, SGM2-5 were immortal, and SGM6 is long-lived, slow-growing, and possibly immortal.

**Figure 6:**
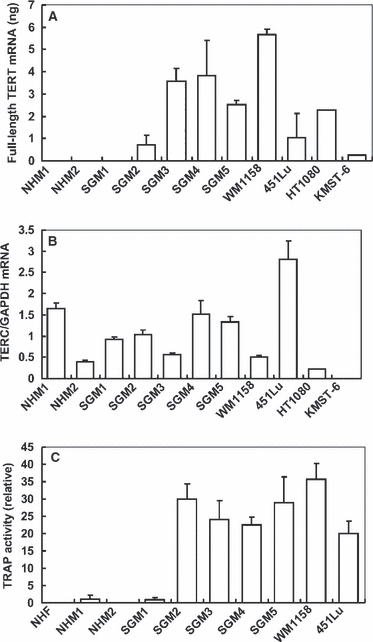
Expression of telomerase subunits and activity in non-immortal and immortal melanocytic cultures. (A) expression of full-length telomerase reverse transcriptase (TERT) from splice variant analysis. Mean and range of duplicates. Qualitatively similar results were obtained by Q-PCR, though with greater variability (not shown). All melanoma lines that expressed full-length TERT also expressed the inactive β-deletion splice variant but little or no dominant negative α-deletion variant (data not shown). (B) Telomerase RNA component expression by Q-PCR. Mean and SEM of triplicate cultures, with duplicate readings from each. (C) telomerase activity by the telomerase repeat amplification protocol assay. Mean and SEM of six readings, three each from two experiments. NHM, normal human melanocytes; NHF, normal human dermal fibroblasts. SGM lines: see Table 3. WM1158 and 451Lu: established metastatic melanoma lines. HT1080 and KMST-6: positive and negative control lines.

To investigate association between immortalization and telomerase, we tested the immortal lines and a selection of non-immortal cultures for telomerase activity by the telomerase repeat amplification protocol (TRAP) assay and for the expression of the two subunits of telomerase, TERT and telomerase RNA component (TERC) (the RNA component), by Q-PCR and also by TERT splice-variant analysis. Two normal melanocyte cultures and the non-immortal RGP-derived culture SGM1 all expressed TERC, but none of these, nor normal fibroblasts, had detectable full-length TERT or telomerase activity (Figure 6). However, all tested immortal lines (SGM2 to SGM5) did display both telomerase activity and full-length TERT as well as TERC expression. Levels were comparable to those seen in control metastatic melanoma lines (Figure 6A,C). Lines positive and negative for full-length TERT by splice-variant analysis were likewise positive and negative, respectively, by Q-PCR (data not shown).

## Discussion

To clarify the basic mechanisms of melanoma initiation, we directly tested the twin hypotheses that nevus cells are senescent and melanoma cells are immortal, using conditions optimized for survival and proliferation of each lesion type. Under these conditions, benign and dysplastic nevi both typically showed a little proliferation and then senesced, displaying three markers of senescence. However, the proliferation of nevus explants as separate colonies suggested that only a small fraction of explanted cells actually grew. This fraction might be contaminating normal melanocytes, although because they were equally common in dermal and epidermal explants, this would require that normal melanocytes commonly enter the dermal part of a nevus. At any rate, division continued for a few passages at most, so even these cells appeared nearly senescent. Moreover, the nevi studied here were not quite typical, having all been excised because of a clinical suspicion of malignancy, although finally diagnosed as nevi. High-passage normal human fibroblast cultures regarded as senescent contained 4–8% of cells able to proliferate (enter S-phase) (Seshadri and Campisi, 1990). If we similarly regard a lesion in vivo as senescent if say <5% of the cells can divide, then even though we saw some proliferating cells, our findings are still consistent with describing nevi as senescent. This accords completely with their static nature and the expression of senescence markers (Gray-Schopfer et al., 2006; Michaloglou et al., 2005).

Given the common belief that immortalization is necessary for malignancy (Collado et al., 2007; Stewart and Weinberg, 2006), we previously hypothesized that immortalization initiates a melanoma (Bennett, 2003; Gray-Schopfer and Bennett, 2006). However, we now report that most primary melanoma cultures – despite initially growing well under the optimized conditions – later arrest. This arrest differs from normal senescence, as senescence markers are less prevalent or faint, and abnormal or multinucleate morphology and cell death seem common. The level of p16 protein expression is frequently low, as seen in uncultured melanoma sections (Gray-Schopfer et al., 2006; Keller-Melchior et al., 1998; Talve et al., 1997). Senescence is known to be defective in p16-deficient melanocytes (Ha et al., 2007; Sviderskaya et al., 2002, 2003), leading in human melanocytes to telomeric crisis in the absence of TERT (Sviderskaya et al., 2003). Thus, an attractive explanation for the arrest, bizarre morphology and cell death in melanoma cultures is telomeric crisis. As a more specific test of crisis, we looked for anaphase bridging and other mitotic abnormalities in uncultured melanoma sections and found that they were indeed common in VGP melanomas.

Abnormal mitoses and pleomorphic cells are long-established indicators of malignancy. However, there is now good evidence to attribute many such mitotic abnormalities, especially anaphase bridging, to telomere dysfunction, implying that these particular cancer cells would be in crisis and not immortal (Artandi and DePinho, 2010; DePinho and Polyak, 2004; Gisselsson and Höglund, 2005; Gisselsson et al., 2001). Specifically, telomeric crisis phenotypes (including anaphase bridging) have been reported to peak at the stage of carcinoma in situ/early adenocarcinoma in human breast and colorectal cancers (Chin et al., 2004; Rudolph et al., 2001) and to be common in overt carcinoma in the pancreas and ovary (Hezel et al., 2006; Höglund et al., 2003). Chin et al. (2004) nicely demonstrated parallels between telomeric crisis in culture and in carcinoma in situ, including a peak in gene copy-number variance. There are other possible mechanisms that can yield generally abnormal mitoses, for example S/G2 or G2/M checkpoint deficiencies, extra centrosomes, or misalignment of chromosomes (Peddibhotla and Rosen, 2009). However, these mechanisms do not give individual anaphase bridges. (Entering mitosis with incompletely replicated DNA would give multiple bridging or no clear anaphase.) Because abnormal mitoses (or any mitoses) are very rare in nevi, and as RGP melanomas also have few mitoses, it can at least be concluded that telomeric crisis is rare in pigmented lesions less progressed than the VGP. From this and the fact that immortalization is unlikely before crisis (there would be no selective pressure for it), the most probable conclusion is that immortalization does not typically occur before the VGP stage. This is consistent with the cell-culture findings and quite comparable to the above-cited evidence for crisis occurring within various early carcinomas rather than before their initiation.

**Figure 7:**
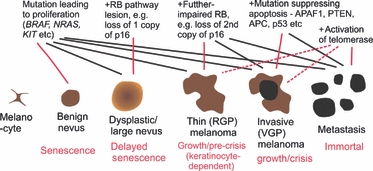
Proposed update of model for melanoma progression. Revised from model of Bennett (2003). Red lines indicate additional genetic changes proposed for each lesion type compared to less progressed lesions. Dashed lines indicate not in all lesions. Benign nevi are still regarded as senescent lesions. Primary melanomas are still assumed to have escaped normal p16/RB-based senescence but are now proposed to be typically pre- or pericrisis rather than immortal. Activation of telomerase is now proposed as a late event, conferring immortality, and (from literature cited) common in metastatic melanoma.

Accordingly, we now propose a revised genetic model of melanoma progression (Figure 7), in which immortalization with TERT expression is not required for melanoma initiation as previously proposed but is associated more with progression. (Note that such a model is only a hypothesis to integrate current evidence, not a firm conclusion.) Proliferating early melanomas would be post-senescence but precrisis, as shown. As RGP melanomas have few mitoses (Mooi and Krausz, 2007) and exhibit localized p16 and/or p21 in vivo (Gray-Schopfer et al., 2006), they may retain some competence to senesce in some areas, but presumably other areas have evaded senescence and are growing, as a non-growing lesion would not be diagnosed as a melanoma.

We have confirmed for the first time a separate prediction of the earlier model, retained here: that RGP cells are keratinocyte-dependent for survival. This is interesting, as a potential explanation for the radial growth pattern itself. Invasive growth could then arise through a genetic or epigenetic alteration suppressing apoptosis, such as loss of APAF1, PTEN, a number of which are common in VGP melanomas (Figure 7) (Bennett, 2008). Alternatively, a microenvironmental change in which host stromal or reactive cells started to produce survival factors for the melanoma cells might achieve the keratinocyte independence.

Mechanisms other than crisis might mediate arrest in melanoma cultures. Perhaps it could have been an artifact of explantation. For example, mouse fibroblast senescence has been attributed to stress by atmospheric oxygen level (Parrinello et al., 2003). However, human diploid fibroblasts still senesce in hypoxic conditions (Chen, 2000). The difference may be connected with the fact that human cells require the activation of telomerase for immortality, unlike mouse cells. Moreover, normal neonatal melanocytes and also adult p16-deficient melanocytes will grow for 50 doublings or more under these conditions (Sviderskaya et al., 2003), while metastatic melanomas frequently do yield immortal cultures at ambient oxygen levels (Hsu et al., 2000; Semple et al., 1982). Thus, a fairly short lifespan at ambient oxygen levels appears to be a typical property of primary melanoma and nevus cells, but not of metastatic melanoma cells and normal melanocytes, revealing a consistent biological difference.

It is possible that this limited lifespan of early melanomas is observed only in cell culture. Quintana et al. (2008, 2010) reported that 30% of all cells from uncultured human melanomas could form tumors if implanted in Matrigel in immunodeficient mice, and where tested, these tumors could be serially transplanted, indicating immortality. However, nearly all these melanomas were either metastases, previous xenografts, or stage III (metastatic) primary melanomas. To date, only one stage II (non-metastatic) melanoma was tested (5/6 groups of 10 cells formed tumors) and no stage I, which includes RGP melanomas (Quintana et al., 2010). Thus, as the authors state, more work is needed to assess the tumorigenic and proliferative potential of early-stage melanoma cells. The findings strengthen the idea that metastatic melanoma cells are typically immortal.

Perhaps immortal cells were sometimes present but were inhibited by our conditions. TPA and SCF inhibit the growth of some metastatic melanomas (Hsu et al., 2000; Zakut et al., 1993), and some immortal VGP lines grow in simpler media than ours (Herlyn et al., 1985a; Hsu et al., 2000; Pope et al., 1979; Semple et al., 1982). We did initially vary the conditions, including omitting growth factors, and observed growth stimulation rather than inhibition by the growth factors. Moreover, the frequency of establishment of melanoma lines from small samples in such simpler media by the highly experienced Wistar group (Hsu et al., 2000) was apparently no higher than ours. Still it remains possible that conditions optimized for growth rate may not be optimal for lifespan. This can be tested in future by explanting melanoma metastases, at least some of which contain immortal cells, in a medium optimized for their initial growth, as well as in the medium used here for VGP cells. Again, melanomas are heterogeneous, and immortal cells might be in the lesions but missing from the fragments cultured. However, we based the sample diagnosis on adjacent sections, so the explant should generally correspond to this diagnosis. The conditions used here were optimized for the bulk population in freshly explanted lesional cultures. Thus, we can conclude that this bulk population is not usually immortal under these conditions. The melanoma lines we did obtain had minimal-deviation karyotypes and phenotypes and seem to provide needed representatives of early stages of oncogenesis.

Can a cancer be non-immortal? This departs from the dogma that cellular immortality is a hallmark of cancer. However, as cited earlier, crisis (preimmortal) phenotypes have been reported before in a range of early human cancers, providing some precedent for the possibility (Hezel et al., 2006; Höglund et al., 2003). Moreover, non-immortal human cells including melanocytes may undergo 50 or more population doublings (Bennett and Medrano, 2002), yielding potentially over a tonne of cells if none died. A mutant cell could thus theoretically proliferate into a typical metastatic tumor bulk without immortalization. However, immortality does appear common in advanced cancers, including metastatic melanoma, perhaps because oncogenes accelerate senescence and/or because many lesional cells do die. We present initial evidence here that this immortalization is associated with progression rather than initiation of melanoma.

## Methods

### Materials

Gibco culture media and FCS were obtained from Invitrogen Ltd, Paisley, UK. NUNC tissue culture plastics were from Fisher Scientific (Loughborough, UK). TPA, CT, mitomycin C, and other reagents were from Sigma-Aldrich Co. (Poole, UK). Human SCF was from R&D Systems Europe (Abingdon, UK) or Invitrogen and EDN1 from Bachem (Weil am Rhein, Germany).

### Primary culture of clinical pigmented lesions

See Data S1 and Soo et al. (in press) for more details. ‘Growth medium’ for lesional cells was RPMI 1640 with penicillin (10^4^ U/ml), streptomycin (100 μg/ml), glutamine (2 mM), extra phenol red (7.5 μg/ml), 10% FCS, 200 nM TPA (20 nM TPA for VGP cells once diagnosed), 200 pM CT, 10 ng/ml SCF, and 10% CO_2_, unless otherwise stated. Mitomycin-inactivated XB2 mouse keratinocytes were plated at ∼5 × 10^5^/ml, 0.5 ml/well, into a 24-well culture plate, in DMEM with 10% FCS. Melanocytic lesions were obtained directly after surgery, with all subjects’ informed consent, from St. George's’ Pigmented Lesions and Plastic Surgery clinics. They were transported in chilled medium to a consultant pathologist for assessment. If permitted, a sliver of tissue was cut for culture, leaving the rest for diagnosis.

Epidermal and dermal portions were separated from the specimen as described in Data S1, dissociated, and plated in growth medium (0.5 ml/well) on to the feeder cells: three wells for the dermal and one for the epidermal suspension. The cultures were incubated at 37°C, with fresh medium twice weekly, and subcultured as described in Data S1.

Established SGM lines were grown in the same growth medium, except that for the VGP line SGM5, RPMI1640 was mixed 1:1 with DMEM and the TPA concentration was 20 nM.

### Other cell cultures

All cultures were grown at 37°C with 10% CO_2_ in air. Normal human melanocytes were from our stocks or Cascade Biologics melanocytes were purchased from Invitrogen Ltd. They were grown as described (Gray-Schopfer et al., 2006), in the above medium plus EDN1 (10 nM). Human metastatic melanoma lines WM1158 and 451Lu were a gift from M. Herlyn and were grown in RPMI 1640 and 10% FCS. Normal human dermal fibroblasts were grown in DMEM and 10% FCS.

### Assays of growth and apoptosis

For growth curves, triplicate hemocytometer counts were taken at subculture. The fold increase since plating was calculated and converted to population doublings, which were plotted cumulatively. For short-term assays of proliferation, cells were plated in triplicate 3-cm dishes in each test medium; media were renewed twice weekly. Cultures were harvested after the stated period; duplicate hemocytometer counts per dish were used to calculate mean and SEM of cell numbers. To assess apoptosis, cells were plated in triplicate 3-cm dishes at 3 × 10^4^ cells/ml and grown with the indicated supplements for about 5 weeks. Cells were harvested with trypsin, chilled, pooled with the medium from the same dish (containing any detached cells), centrifuged, and resuspended in PBS containing 1% FCS. They were counted by hemocytometer and resuspended at 5 × 10^4^ cells/ml. They were spun on to glass slides in a Cytospin unit (Universal 320, Hettich Zentrifugen; GMI Inc., Ramsey, MN, USA), air-dried, fixed in 4% formaldehyde, washed twice in calcium- and magnesium-free Dulbecco's PBS (PBSA; pH 7.2), post-fixed in 2:1 ethanol:acetic acid at −20°C, and washed twice in PBSA. Terminal deoxynucleotidyl transferase dUTP nicked-end labeling (TUNEL) staining was performed with an Apoptag kit using fluorescein detection (Millipore, Billerica, MA, USA), according to the manufacturer's instructions. Nuclei were counterstained with Hoechst 33258, specimens mounted in Citifluor antifade medium (Citifluor Ltd, Leicester, UK), and apoptotic cells counted by two different observers, whose counts were similar and were averaged for each slide.

### β-galactosidase staining

In subdued light, 1 mg/ml X-gal solution (5-bromo-4-chloro-3-indolyl-β-galactopyranoside; Sigma-Aldrich Co), 0.12 mM K_3_Fe(CN)_6_, 0.12 mM K_4_Fe(CN)_6_, and 1 mM MgCl_2_ in PBSA, pH 7.0, were prepared. Cells were washed in PBS (complete Dulbecco's PBS with CaCl_2_ and MgCl_2_, pH 7.2) and fixed with fresh 0.5% glutaraldehyde in PBS for 5 min at room temperature. Washed again, the cells were stained with X-gal solution (pH 7.0) for 24 h at 37°C in air. The pH is modified from Dimri et al. (1995) because growing melanocytes also often stain at pH 6.0; Gray-Schopfer et al., 2006). Senescent normal human melanocytes were the positive control. Cells were viewed in water and photographed using an Olympus IMT-2 inverted microscope (Olympus, Southend-on-Sea, UK).

### Immunofluorescence

Cells were grown on glass coverslips in a 4-well plate, or primary cultures in their original 24-well plate were cut out using a heated scalpel. Subconfluent cells were washed in PBS. They were fixed with fresh 3.7% formaldehyde in PBS for at least 20 minutes and permeabilized with 0.5% Triton X-100 for 5 min [except fixation was with fresh 4% paraformaldehyde for H3M3K9 staining]. After blocking with 10% FCS in PBSA for 1.5–2 h, cells were incubated with primary and FITC-conjugated secondary antibodies in 1% BSA in PBSA at room temperature for 1 h each. Antibodies and concentrations were as follows: mouse anti-p16 (NeoMarkers, Newmarket, UK) (1:50) with anti-mouse IgG (FITC) (Sigma-Aldrich) (1:50) and rabbit anti-histone H3 trimethylated on lysine 9 (H3M3K9) (ab8898; Abcam plc, Cambridge, UK) (1:5000) with goat anti-rabbit IgG (FITC), (ab6717; Abcam) (1:200). Cells were washed three times (5 min) in complete PBS and counterstained with Hoechst 33258 (0.2 μg/ml for 10 min). They were rinsed in water and mounted in Citifluor. Photography was with an Axioplan 2 fluorescence microscope and AxioVision 3 digital imaging (Carl Zeiss Ltd, Welwyn Garden City, UK).

### *BRAF* and *NRAS* genotyping

Amplification of *BRAF* exon 15 and *NRAS* exon 2 and 3 was performed using the following primers (20 μM): *BRAF* exon 15: forward, TCATAATGCTTGCTCTGATAGGA and reverse, GGCCAAAAATTTAATCAGTGGA. *NRAS* exon 2: forward, GAACCAAATGGAAGGTCACA and reverse, TGGGTAAAGATGATCCGACA. *NRAS* exon 3: forward, CAATAGCATTGCATTCCCTGTG and reverse, CTAGTGTGGTAACCTCATTTCCC. PCR products were electrophoresed through a 2% agarose gel containing ethidium bromide (50 μg/ml) at 100 V for 40 min. The appropriate bands were excised, and gel purification was performed using a Quick Clean DNA gel extraction kit (Qiagen) as per instructions. DNA was eluted in 20 μl of TE buffer. Automated DNA sequencing was performed using the same primers (3.2 pmol).

### TERT mRNA and TERC expression analysis

Q-PCR (Atkinson et al., 2005) was carried out in triplicate using the Opticon 2 DNA engine and software (MJ Research Inc., Waltham, MA, USA). SYBR green was the fluorophore. TERC (hTR) expression was quantitated using the primers: TRC3F, 5′- TAACCCTAACTGAGAAGGGCGTA-3′ and TRC3R, 5′-GGCGAACGGGCCAGCAGCTGACATT-3′. It was normalized to glyceraldehyde-3-phosphate dehydrogenase (GAPDH) mRNA (primers: GAPDH0.45F: 5′-ACCACAGTCCATGCCATCAC-3′ and GAPDH0.45R: 5′-CCACCACCCTGTTGCTGTA-3′). TERT full-length expression was measured against a standard curve generated using the pBABE vector with a TERT cDNA insert (genomic DNA was used as standard for all other Q-PCR reactions) (primers: TERTFULLF: 5′-CATCCCCCAGGACAGGC-3′ and TERTFULLR: 5′-CTGTCAAGGTAGAGACGTGGCT-3′). This was then normalized to GAPDH mRNA level. TERT splice-variant expression was analyzed according to Keith and Hoare (2004), using primers that detect all splice variants (primers: HT2026F 5′-GCCTGAGCTGTACTTTGTCAA-3′ and HT2482R 5′-GCCAAACAGCTTGTTCTCCATGTC-3′). Products for the four main splice variants (full length, α-deletion, β-deletion, and αβ-deletion) were analyzed using the Agilent Bioanalyser 2100 and DNA-1000 assay chips (Agilent Technologies, Santa Clara, CA, USA).

### Telomerase repeat amplification protocol assays

The method was according to Bilsland et al. (2009). The TRAPeze XL kit was used, following the manufacturer's instructions (Millipore, Watford, UK). Cell pellets were lysed in CHAPS buffer and protein concentration was estimated using the Bio-Rad assay (BioRad Laboratories Ltd, Hemel Hempstead, UK). A concentration of 0.5 μg protein was mixed with the TRAPeze reaction mix containing the TS primer, fluorescein-labeled RP primer, control template, and sulforhodamine-labeled control K2 primer. Each assay included no-telomerase, no-Taq, and heat-treated controls. Extension products were generated at 30°C, followed by Q-PCR detection in triplicate using the Chromo4 equipment and software (BioRad). Total product generated was measured against the TR8 standards. (The internal control was used to check that PCR was not inhibited, not for normalization.)

## Supporting information

**Data S1.** Detailed primary culture method for pigmented lesions.

**Figure S1.** Changing morphology of a benign nevus culture.

**Figure S2.** Additional examples of p16 immunostaining of arrested lesional cultures.

**Figure S3.** Additional examples of H3M3K9 and β-galactosidase immunostaining of arrested cultures.
