# Supplementary material for: Malignancy without immortality? Cellular immortalization as a possible late event in melanoma progression
Source: Pigment Cell Melanoma Res. 2011 Mar 21;24(3):490–503. doi: 10.1111/j.1755-148X.2011.00850.x (PMC3123747; doi:10.1111/j.1755-148X.2011.00850.x)
Supplement: Supplementary file 1 [file pcmr0024-0490-SD4.doc]

**Supplementary Information: Soo et al.**

***Contents:***

**Detailed primary culture method for pigmented lesions 1**

**Supplementary figure legends - - - - 3**

**Supplementary figures 1-3 - - - - - 4-6**

**Detailed primary culture method for pigmented lesions**

Growth factor stocks were dissolved in PBS with 1 mg/ml BSA as carrier, and aliquots stored at -70°C. TPA was dissolved in ethanol and stored at -70°C; the working stock was in PBS and BSA as above, also stored at -70°C. “Growth medium” for lesional cells was RPMI 1640 with penicillin (10^4^ U/ml), streptomycin (100 µg/ml), glutamine (2 mM), extra phenol red (7.5 µg/ml added), 10% FCS, 200 nM TPA, 200 pM CT and 10 ng/ml SCF, pH ~7.0 in 10% CO_2_, except that 20 nM TPA was eventually used for VGP melanoma cells.

Mouse keratinocytes of the XB2 immortal line [1] were mitomycin-inactivated for use as feeder cells, and stocks frozen as described [2], except that cells were inactivated with 8 µg/ml mitomycin C for 3-3.5 hr. They were thawed and plated at ~ 5 x 10^5^/ml, 0.5 ml/well, into 4 wells of a 24-well culture plate per lesion, 4 to 48 hours in advance, in DMEM medium with 10% FCS.

Melanocytic lesions were obtained with informed consent and ethical approval, directly after surgery, from the Pigmented Lesions and Plastic Surgery clinics, St George’s Healthcare Trust. They were placed in a sterile, screw-topped container of chilled RPMI 1640 with antibiotics and glutamine but no serum, on ice, and taken to a consultant pathologist who assessed the lesion. If the lesion was suitable and large enough, a sliver of tissue was cut from the edge and placed in fresh medium, leaving the rest for diagnosis. Importantly, diagnoses reported here are based on sections adjacent to the portion cultured, rather than the overall lesion diagnosis.

The sample was placed sterilely on a 35-mm culture dish and rinsed in PBSA (calcium and magnesium-free Dulbecco’s PBS, pH 7.2). On a dissecting microscope in a laminar flow hood, fat and reticular dermis were removed using sterile forceps and a scalpel with curved blade. In a fresh dish, the specimen was covered with trypsin (250 μg/ml) and EDTA (200 μg/ml) in PBSA, and optionally stored at 4°C overnight. The specimen was placed at 37°C in air for 15 minutes, or until the epidermis could easily be detached. Using the microscope, the epidermis was gently removed, placed in a dish, covered in the same trypsin solution and chopped finely with two scalpels. Residual reticular dermis was separated from the dermal portion and discarded. The papillary dermis was placed in a new 35-mm dish, covered in fresh trypsin-EDTA, chopped finely and incubated at 37°C in air for 45 minutes. The preparations were pipetted, transferred to microcentrifuge tubes, centrifuged at 2000 rpm for 5 minutes, and the supernatant aspirated. Complete growth medium (see above) with soybean trypsin inhibitor (125 µg/ml), was used to resuspend the pelletted cells, which were plated (0.5 ml/well) on to the prepared feeder cells, immediately after removal of most of the DMEM medium. Three wells were used for the dermal and one for the epidermal preparation. The cultures were maintained in a humidified 37°C incubator with 10% CO_2_, with fresh growth medium twice weekly. In general, apparently pure cultures of lesional cells were obtained. These were subcultured if they grew sufficiently. For subculture, cells were rinsed once gently in PBSA and incubated with trypsin (only 125 µg/ml) and EDTA (200 µg/ml) in PBSA until most cells were detached, then resuspended in medium and 10% FCS, counted by hemocytometer and replated in growth medium at 3-5 x 10^4^ cells/ml if possible, or on new XB2 feeder cells if there were insufficient melanocytic cells.

References

[1] Rheinwald JG, and Green H (1975). Formation of a keratinizing epithelium in culture by a cloned cell line derived from a teratoma. *Cell* **6,** 317-330.

[2] Bennett DC, Cooper PJ, Dexter TJ, Devlin LM, Heasman J, and Nester B (1989). Cloned mouse melanocyte lines carrying the germline mutations albino and brown: complementation in culture. *Development* **105,** 379-385.

**Supplementary Figure 1**. Changing morphology of a benign nevus culture.

Cells bipolar and only slightly flattened at passage 2; senescent-looking (large and multipolar) at passage 4.

**Supplementary Figure 2.** Additional examples of p16 immunostaining of arrested lesional cultures. Various locations of label are seen, including predominantly nuclear (N), predominantly cytoplasmic (C), and little or no staining (0). Other labels as in Figure 4. Melanoma cells (as in vivo) often have cytoplasmic p16 only, suggesting inactivity, or else little or no p16. Only nuclear p16 was scored in Table 1B, but there may have been false positives, as it is sometimes difficult to distinguish faint nuclear p16 from cytoplasmic p16.

**Supplementary Figure 3.**  Additional examples of H3M3K9 and β-galactosidase immunostaining of arrested cultures. Labels as in Figure 4. (Fib): senescent human dermal fibroblasts, showing specificity of the antibody. Prominent heterochromatic foci (SAHF) are seen in the nuclei. (Note: imaged by normal rather than confocal microscopy, so the SAHF appear clustered. SAHF are not seen in the Hoechst panel because lower concentration of dye is required to show these.) Arrested lesional cells showed a pattern of larger heterochromatic foci superimposed on many smaller ones (clearest in the magnified VGP nucleus, seen by Z-stack imaging, 100x objective). Bottom panel: β-galactosidase staining may be partly obscured by substantial melanin pigmentation in arrested cultures.
