# Supplementary figures and images for: Malignancy without immortality? Cellular immortalization as a possible late event in melanoma progression
Source: Pigment Cell Melanoma Res. 2011 Mar 21;24(3):490–503. doi: 10.1111/j.1755-148X.2011.00850.x (PMC3123747; doi:10.1111/j.1755-148X.2011.00850.x)

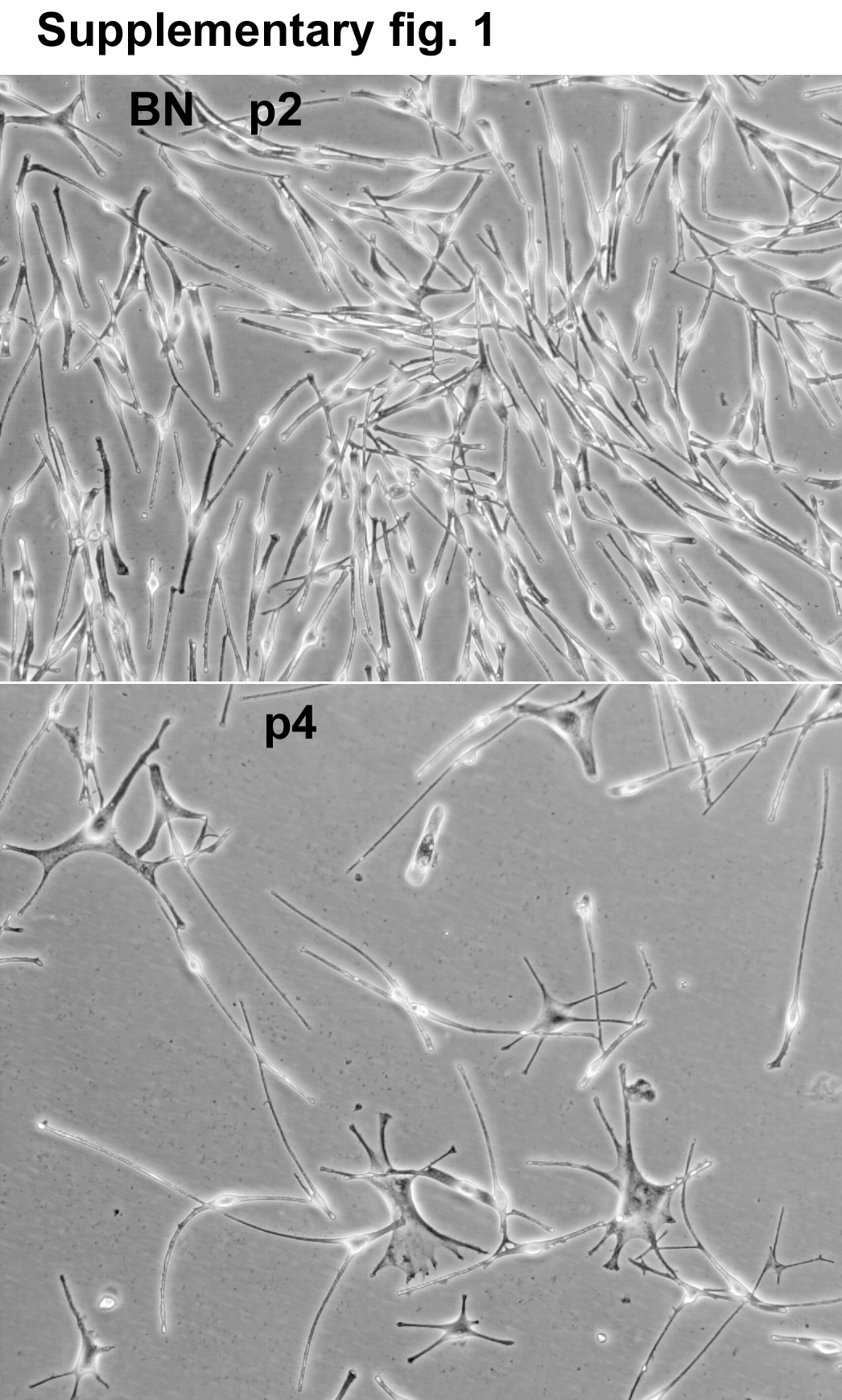

Supplement: Supplementary file 2 [file pcmr0024-0490-SD1.tif]

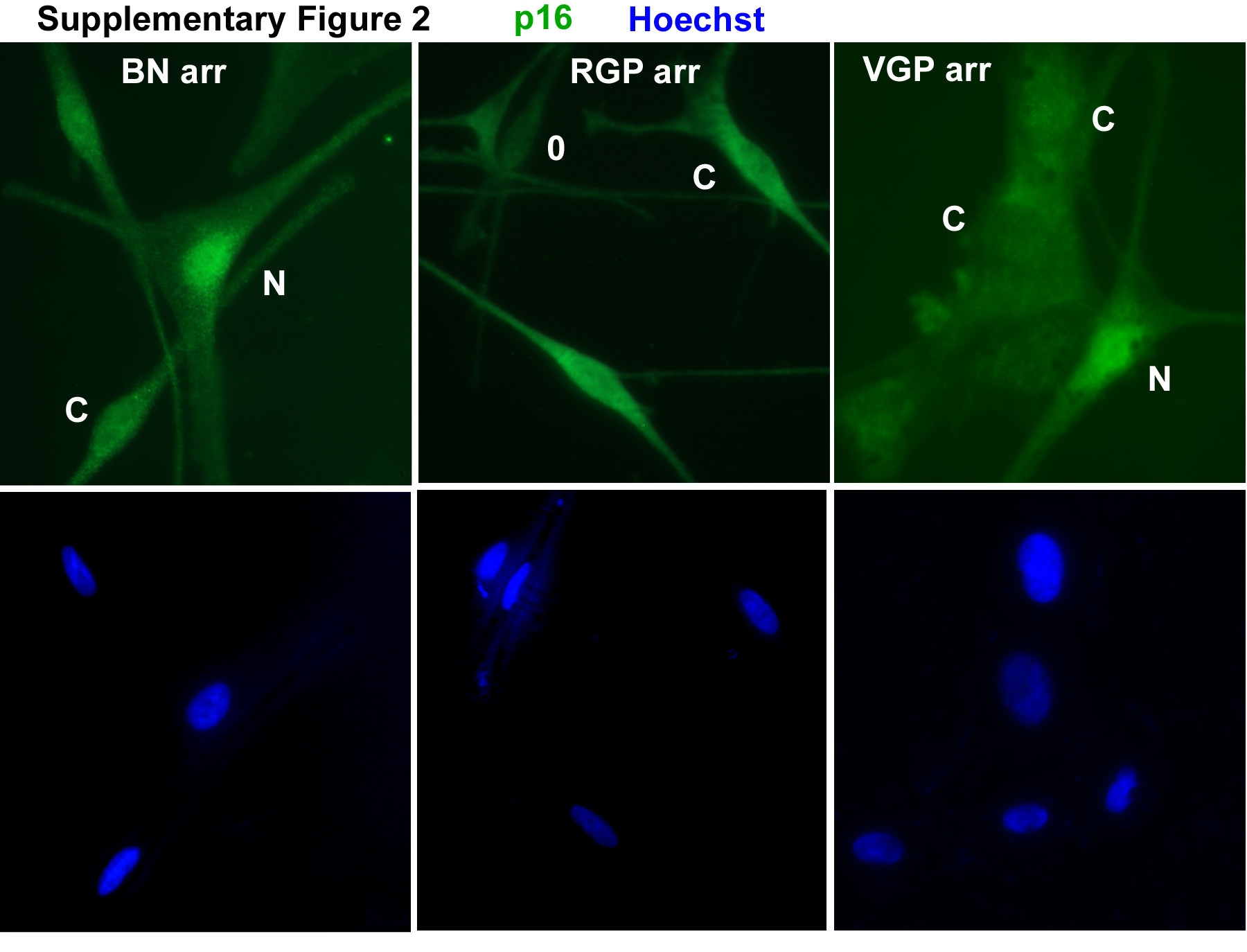

Supplement: Supplementary file 3 [file pcmr0024-0490-SD2.tif]

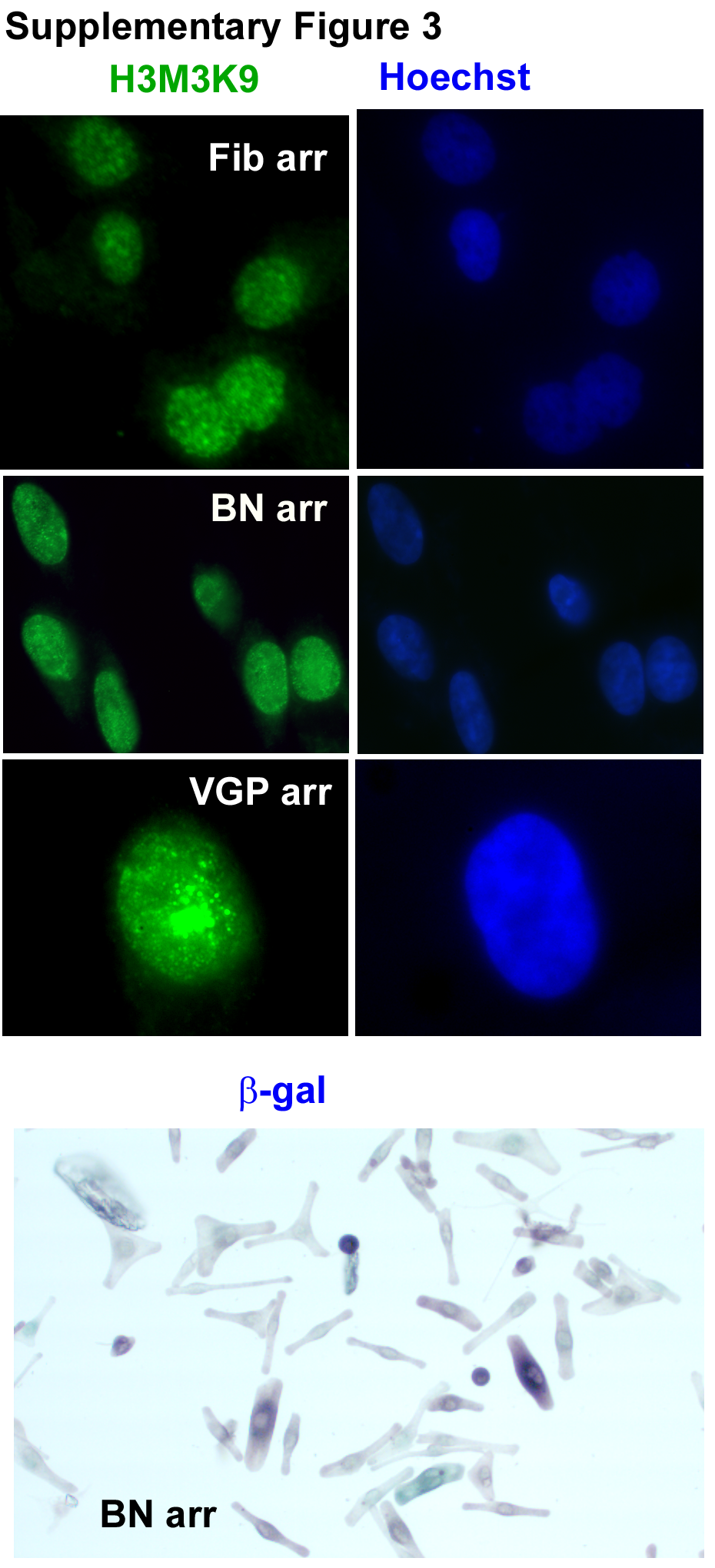

Supplement: Supplementary file 4 [file pcmr0024-0490-SD3.tif]
